# The protective power of dissent? A longitudinal study on cognitive and socio-emotional determinants of COVID-19 vaccine hesitancy among young people in Canada

**DOI:** 10.1177/13634615241296293

**Published:** 2024-11-18

**Authors:** Diana Miconi, Anna Levinsson, Mohammed Abdullah Heel Kafi, Cindy Ngov, Tara Santavicca, Cécile Rousseau

**Affiliations:** 1 Department of Educational Psychology and Adult Education, 5622 Université de Montréal, Montreal, QC, Canada; 2 Centre de Recherche du Centre Hospitalier de l’Université de Montréal – CRCHUM, Montréal, QC, Canada; 3 Department of Epidemiology, Biostatistics and Occupational Health, 5620 McGill University, Montréal, QC, Canada; 4 Department of Social Medicine and Public Health, Sahlgrenska Academy at Gothenburg University, Gothenburg, Sweden; 5 507266 McGill International TB Centre, RI-MUHC, Montreal, QC, Canada; 6 Research and Action on Social Polarisations (RAPS), Montréal, QC, Canada; 7 Division of Social and Cultural Psychiatry, 5620 McGill University, Montréal, QC, Canada

**Keywords:** conspiracy beliefs, COVID-19, dissent, psychological distress, vaccine hesitancy

## Abstract

COVID-19 has elicited polarized reactions to public health measures, fueling anti-vaccination movements worldwide which indicate that vaccine hesitancy represents a common expression of dissent. We investigate changes in cognitive (i.e., trust in government, conspiracy beliefs, vaccine attitudes, and other COVID-19-related factors) and socio-emotional factors (i.e., psychological distress and social support) over time, and examine if these factors are associated with COVID-19 vaccine hesitancy. A sample of Canadian young adults (*N* = 2,695; 18 to 40 years old) responded to an online survey in May/June 2021 (after the first vaccination campaign) and then in November 2021 (after vaccine mandates were introduced). Based on survey answers, participants were categorized as “not hesitant”, “hesitant”, and “do not intend to get vaccinated” at each time point. Results from generalized estimating equation models indicate that vaccination hesitancy decreased over time. The importance attributed to specific COVID-19-related factors (e.g., research and science about COVID-19 vaccines, opinions of friends and family) decreased whereas psychological distress increased over time. Cognitive and socio-emotional factors were associated with vaccine hesitancy, with participants who did not intend to get vaccinated reporting the lowest psychological distress scores. We argue that dissent may be an empowering way for young people to restore a sense of personal agency via the opposition to a system perceived as illegitimate and/or unfair. These results raise important questions about potential collateral effects of top-down government and public health interventions in times of crisis.

Disinformation spread rapidly and globally during COVID-19, and anti-vaccination movements promoting protest and disobedience increased in popularity by suggesting that vaccination was a major threat to life and/or individual freedom (Flaherty et al., 2021; Verger & Dubé, 2020). Social media use was found to increase significantly during crisis situations where fast information flow is needed, including during the COVID-19 pandemic (Puri et al., 2020). Although the use of social media can represent an opportunity during health emergencies to easily access up-to-date information, it also represents a potential source of misinformation at both the local and global levels which can fuel vaccine hesitancy (Puri et al., 2020; Wilson & Wiysonge, 2020). Indeed, the rapid spread of anti-vaccine campaigns online has been extensively documented during the COVID-19 pandemic (Burki, 2020). Public discourses online and offline labeled “anti-vaxxers” as “self-centered”, “immoral”, or even “stupid” and “criminal” because their resistance to public health measures was perceived as a threat to collective safety (Labbé et al., 2022). To date, there has been limited examination of the heterogeneity of individuals who oppose vaccination (Bardosh et al., 2022; Santavicca, Ngov, et al., 2022) and a very limited exploration of the possibility that refusing vaccines represents a form of dissent and critique of a social order perceived as unfair (Bergem, 2022; Mucchielli, 2020; Smith et al., 2023). From this perspective, not following vaccine mandates, beyond being a personal health decision, could represent a coping strategy to regain power, a voice, and a sense of community to counter feelings of helplessness during uncertain and socially polarized times (Williams et al., 2021). The evolution of cognitive and socio-emotional factors associated with vaccine hesitancy (i.e., the delay or refusal to be vaccinated) during the pandemic has yet to be investigated. Following public health and governmental vaccination guidelines is the result of a complex decision-making process guided not just by cognitive, rational beliefs, but also by socio-emotional and political processes at the individual and collective levels that can lead to inaccurate beliefs and biases (Attwell et al., 2022; Dubé & Macdonald, 2022; Lerner et al., 2015). These processes vary over time and influence both pro-vaccine policymakers and their opponents, and both sides can legitimately argue that the other is hiding or manipulating the truth, leaving little space for nuances or compromises (Bardosh et al., 2022). From both sociological and political perspectives, it is important to go beyond moral and public health discourses to deepen our understanding of determinants of dissent with empirical evidence. This article focuses on COVID-19 vaccine hesitancy as a potential form of dissent in times of increased social polarization. Specifically, we investigate longitudinal associations between cognitive and socio-emotional factors and COVID-19 vaccine hesitancy among young people from three Canadian provinces.

## Epidemiology of COVID-19 vaccine hesitancy

Numerous studies have investigated socio-demographic, cognitive, and socio-emotional determinants of COVID-19 vaccine hesitancy with the ultimate aim to inform strategies to increase vaccine uptake and reduce infections, hospitalizations, and deaths related to COVID-19. Notably, the pandemic literature relies mostly on cross-sectional studies that were conducted early during the pandemic (often before the availability of COVID-19 vaccines) and has tried to circumscribe the numerous determinants of vaccine hesitancy. In light of the upsurge in false information and conspiracy beliefs around the COVID-19 virus and vaccines, there is a body of literature on cognitive determinants of COVID-19 vaccine hesitancy (Limbu & Gautam, 2023; Mulukom et al., 2022; Oleksy et al., 2022). Negative attitudes toward vaccines in general, specific socio-cultural beliefs toward COVID-19, lack of trust in government and authorities, as well as conspiracy beliefs have been suggested to drive vaccine hesitancy across multiple studies (Cénat et al., 2020; Limbu & Gautam, 2023; Loomba et al., 2021; Moscardino et al., 2022; Mulukom et al., 2022; Santavicca, Ngov, et al., 2022). In terms of socio-emotional factors, evidence suggests that vaccine hesitancy is associated with lower levels of perceived social support from friends and family (Moscardino et al., 2022) and lower levels of neighbourhood support (Jaspal & Breakwell, 2022). Authors argue that social support promotes a sense of connectedness and facilitates information gathering, feelings of self-efficacy, and engagement in preventive action. However, no association between social support and vaccine hesitancy was detected in a study focused on the Canadian context (Santavicca, Ngov, et al., 2022). Country specificities in terms of socio-cultural habits and/or public health measures may be partly responsible for conflicting findings. Specific to psychological distress, studies have not found an association between depression/anxiety and vaccine hesitancy (Bendau et al., 2021; Chaudhuri et al., 2022; Moscardino et al., 2022; Santavicca, Ngov, et al., 2022).

Although vaccine mandates in many countries including Canada have been supported by the majority of the population (Neilson, 2021), they have simultaneously provoked considerable social and political unrest among both the vaccinated and non-vaccinated (Bardosh et al., 2022; Beddoe, 2022; Lord, 2021; Lyu et al., 2022). Indeed, the scientific evidence for mandatory vaccine policies has been increasingly challenged due to repeated shifts in communicated public health rationales (e.g., from herd immunity and back to “normal” to reducing the burden on the health care system), as well as waning effectiveness of COVID-19 vaccines in limiting transmission and infections (Bardosh et al., 2022). Pandemic fatigue and prolonged social distancing, as well as the polarizing reactions to vaccine mandates in many countries, may have influenced endorsement of conspiracy beliefs, attitudes towards vaccines, trust in government, psychological distress, and perceived social support (Krakowczyk et al., 2022). If and how these changes are associated with dissent in terms of vaccine hesitancy has yet to be determined. Another limitation of available research is that most studies included broad age ranges, failing to address the specificities which characterize the period of young adulthood (18–40 years). This age group, invested in exploration of social and personal options, has shown more resistance to vaccination (King et al., 2021; Lavoie et al., 2022; Savoia et al., 2022) and has experienced more adverse mental health consequences of this health emergency than older age groups (Ma et al., 2021; Marques de Miranda et al., 2020; McGinty et al., 2020; National Center on Health Statistics, 2022; Panchal et al., 2021).

## Vaccine hesitancy from a collective and individual psycho-social perspective

As is historically the case, collective and individual benefits (e.g., herd immunity, protection from infection) as well as the personal cost of vaccination (e.g., loss of individual agency and freedom) have been instrumentalized by a range of political and ideological discourses both supporting and resisting vaccination and vaccine mandates (Bilewicz & Soral, 2022; Farias & Pilati, 2021; Till & Niederkrotenthaler, 2022). At the collective level, ethnographic research indicates that vaccine refusal may represent a means of expressing social and political affiliation (Bergem, 2022). In this sense, non-vaccinated minority groups broke the social isolation associated with social distancing, proposing a sense of community and support through the adhesion to dissenting views (Smith et al., 2023). In addition, evidence suggests that policies that restricted freedom and reduced the opportunity of engaging in collective mobilization were associated with an increase in dissent activities during COVID-19 (Schmelz & Bowles, 2021; Wood et al., 2022).

At the individual level, during the pandemic young people have reported perceiving fewer direct benefits from vaccination (Chaudhuri et al., 2022; Daly et al., 2020; Mills & Rüttenauer, 2022), while individuals who were vaccinated because of multiple pressures (e.g., vaccine mandates), despite their own hesitancy, may have experienced higher cognitive dissonance (Bardosh et al., 2022). This may be true in particular when official announcements that vaccination would ensure a return to a “normal life” were followed instead by the adoption of more restrictive and coercive measures (Bardosh et al., 2022).

On the one hand, vaccination may be protective for one's mental health and may promote more robust social networks by reducing risk of infection, negative experiences of stigmatization, and social exclusion (Labbé et al., 2022). Yet, on the other hand, when individual or collective benefits are not clear, vaccination may increase cognitive dissonance and reduce personal agency and social action, all factors that are associated with increased psychological distress (Cooper, 2019; Lundström, 2022). More research is warranted on determinants of vaccine hesitancy to shed light on mechanisms of dissent in the context of a health emergency in socially polarized times.

## The current study

The present study examines factors associated with dissent, using vaccine hesitancy as a proxy, during the COVID-19 pandemic among young adults in Canada. We investigate cognitive (i.e., trust in government, conspiracy beliefs, attitudes towards vaccines, other beliefs around COVID-19) and socio-emotional (i.e., psychological distress and social support) determinants of vaccine hesitancy among young Canadians at two time points over a 6-month period (May 2021 to November 2021) during which vaccine mandates were introduced.

The study was conducted in three of the Canadian provinces that were most hard-hit by the pandemic, namely Alberta, Ontario, and Quebec. The first data collection took place between May 21 and June 14, 2021, a period during which the reported number of COVID-19 cases rapidly decreased as the three provinces ramped up their respective vaccination campaigns and vaccine milestones were reached (CIHI, 2022; Public Health Agency of Canada [PHAC], 2022). Provincial and federal regulations and sanitary measures were changing frequently with various openings and closings of schools and businesses (Labbé et al., 2022). The second data collection took place in November 2021. From July 2021 to November 2021, as the number of COVID-19 cases decreased (PHAC, 2022), social distancing and mask mandates were eased in all three provinces and there was a sharp decrease in the number of announcements issued by the governments (CIHI, 2022). During this period, groups of residents publicly expressed disagreement with official public health policy through protests. For example, when provincial vaccine passport mandates were implemented in September in all three provinces, discontented residents reciprocated with an increase in protests (Austen, 2021; De Castillo, 2021; Ferah, 2021; Rocca, 2021).

We expected vaccine hesitancy to decrease over time in line with rising vaccination rates in Canada at the time of the study (PHAC, 2022), and cognitive factors (lower trust in government, conspiracy beliefs, negative attitudes towards vaccines, and other COVID-19-related factors) to be associated over time with dissent. Given the rapidly evolving social context as well as the scarce or mixed findings in the literature, we did not formulate specific hypotheses on the evolution of determinants of vaccine hesitancy over time, nor on the longitudinal associations between socio-emotional factors (i.e., social support and psychological distress) and vaccine hesitancy.

## Methods

### Participants

A total of 2,695 participants (*M_age _*= 29.6, *SD_age _*= 5.51; 59.6% women) responded to an online survey in both May/June and November 2021. Descriptive statistics of participants are reported in Table 1 (see Supplemental Material for a comparison of descriptive statistics between “not hesitant”, “hesitant”, and “do not intend to get vaccinated” participants at T1).

**Table 1.** Descriptive statistics for *N* = 2,695 individuals who responded to the survey at both time points, separately for T1 and T2.

	T1 (*N* = 2,695)	T2 (*N* = 2,695)
Age (years)		
Mean (*SD*)	29.6 (5.51)	30.0 (5.51)
Gender*		
Woman	1605 (59.6)	1605 (59.6)
Man	1073 (39.8)	1073 (39.8)
Transgender or gender-diverse	10 (0.4)	10 (0.4)
Missing (%)	7 (0.3)	7 (0.3)
Province*		
Alberta	599 (22.2)	599 (22.2)
Ontario	1128 (41.9)	1128 (41.9)
Quebec	968 (35.9)	968 (35.9)
Immigrant generation*		
First	492 (18.3)	492 (18.3)
Second	633 (23.5)	633 (23.5)
Third or more	1559 (57.8)	1559 (57.8)
Missing (%)	11 (0.4)	11 (0.4)
Religiosity*		
No religion	1338 (49.6)	1338 (49.6)
Religious	1239 (46.0)	1239 (46.0)
Missing (%)	118 (4.4)	118 (4.4)
Marital status		
Never married	1340 (49.7)	1280 (47.5)
Divorced/separated/widowed	67 (2.5)	73 (2.7)
Married/common law/living together	1270 (47.1)	1321 (49.0)
Missing (%)	18 (0.7)	21 (0.8)
Education*		
None/Less than high school	45 (1.7)	45 (1.7)
High school graduate	399 (14.8)	399 (14.8)
Apprenticeship, technical institute, trade or vocational school (any year)	161 (6.0)	161 (6.0)
College, CEGEP or other non-university certificate or diploma (any year)	573 (21.3)	573 (21.3)
University certificate, diploma or degree (any year)	1504 (55.8)	1504 (55.8)
Missing (%)	13 (0.5)	13 (0.5)
Income*		
$19,999 or less	144 (5.3)	144 (5.3)
Between $20,000 and $39,999	335 (12.4)	335 (12.4)
Between $40,000 and $59,999	380 (14.1)	380 (14.1)
Between $60,000 and $79,999	401 (14.9)	401 (14.9)
Between $80,000 and $99,999	416 (15.4)	416 (15.4)
$100,000 or more	741 (27.5)	741 (27.5)
Missing (%)	278 (10.3)	278 (10.3)
Psychological distress		
Mean (*SD*)	1.69 (0.567)	1.74 (0.631)
Missing (%)	156 (5.8)	204 (7.6)
Trust in government		
Mean (*SD*)	2.65 (1.10)	2.69 (1.12)
Missing (%)	59 (2.2)	70 (2.6)
COVID-19 conspiracy theory 1**		
Mean (*SD*)	1.99 (1.21)	1.94 (1.22)
Missing (%)	137 (5.1)	139 (5.2)
COVID-19 conspiracy theory 2**		
Mean (*SD*)	1.66 (1.11)	1.66 (1.11)
Missing (%)	132 (4.9)	113 (4.2)
COVID-19 conspiracy theory 3**		
Mean (*SD*)	1.61 (1.05)	1.62 (1.04)
Missing (%)	199 (7.4)	174 (6.5)
COVID-19 conspiracy theory 4**		
Mean (*SD*)	2.12 (1.26)	2.08 (1.27)
Missing (%)	106 (3.9)	104 (3.9)
Social support (total)		
Mean (*SD*)	42.0 (10.6)	41.5 (10.6)
Missing (%)	100 (3.7)	99 (3.7)
Vaccine attitudes (VAX)		
Mean (*SD*)	3.26 (1.25)	3.28 (1.34)
Missing (%)	314 (11.7)	323 (12.0)
Vaccine hesitancy		
Does not intend to get vaccinated	148 (5.5)	111 (4.1)
Hesitant	333 (12.4)	94 (3.5)
Not hesitant	2167 (80.4)	2431 (90.2)
Missing (%)	47 (1.7)	59 (2.2)
Vaccine factor 1***		
Mean (*SD*)	3.95 (1.64)	3.96 (1.71)
Missing (%)	77 (2.9)	108 (4.0)
Vaccine factor 2***		
Mean (*SD*)	4.38 (1.75)	4.11 (1.78)
Missing (%)	67 (2.5)	100 (3.7)
Vaccine factor 3***		
Mean (*SD*)	4.68 (1.57)	4.54 (1.62)
Missing (%)	73 (2.7)	104 (3.9)
Vaccine factor 4***		
Mean (*SD*)	4.10 (1.70)	3.96 (1.70)
Missing (%)	54 (2.0)	100 (3.7)
Vaccine factor 5***		
Mean (*SD*)	5.38 (1.49)	5.37 (1.50)
Missing (%)	51 (1.9)	86 (3.2)
Vaccine factor 6***		
Mean (*SD*)	4.85 (1.53)	4.71 (1.55)
Missing (%)	74 (2.7)	102 (3.8)
Vaccine factor 7***		
Mean (*SD*)	4.10 (1.70)	4.12 (1.68)
Missing (%)	72 (2.7)	98 (3.6)
Vaccine factor 8***		
Mean (*SD*)	3.62 (1.73)	3.62 (1.73)
Missing (%)	71 (2.6)	117 (4.3)

*Note.* * = variable values from T1 were carried over to T2; ** = COVID-19 conspiracy theories 1: “The government is misleading the public about the cause of the Coronavirus”, 2: “The spread of the Coronavirus is a deliberate attempt by a group of powerful people to gain control”, 3: “Coronavirus is a bioweapon developed by China to destroy the West”, and 4: “The mainstream media is deliberately feeding us misinformation about the Coronavirus and lockdown”; *** = Vaccination factors 1: “The current politics”, 2: “The rushed/fast-tracked research and development timeline”, 3: “The frequently changing science of COVID-19”, 4: “Actions and opinions of my friends and family regarding the vaccine”, 5: “My trust in scientists”, 6: “My own reading and research on COVID-19 vaccines”, 7: “The country in which a vaccine is manufactured”, 8: “The potential cost of a coronavirus (COVID-19) vaccine”. *SD* = standard deviation.

### Procedure

This is a two-wave longitudinal study. An online survey using a self-administered Computer-Assisted Web Interface (CAWI) approach was conducted among Canadians 18 to 40 years old in the provinces of Alberta, Ontario, and Quebec using the Leger360 pool of registered members, accessing 500,000 Canadian professionals and consumers (Leger 360, 2022). A total of 50,845 participants were randomly selected by Leger Marketing and received an invitation via email with a private link where they could respond to the survey in English or French, taking approximately 12 minutes to complete. The first data collection took place between May 21 and June 14, 2021 (*n* = 5,007). The second data collection took place between November 1 and November 29, 2021. In November, invitations were sent to 4,609 out of 5,007 participants who participated in the first data collection and 2,695 participants (58.48%) responded to the survey at both data collections. The study was approved by the Institutional Review Board of McGill University.

### Measures

*Outcome: Vaccine hesitancy* was measured using responses to two questions focusing on current vaccination status and COVID-19 vaccination intentions (C4R Investigators, 2020). Participants were grouped into three outcome categories inspired by the vaccine hesitancy spectrum (Dubé et al., 2013): (1) “non-hesitant” if they had already been vaccinated against COVID-19 or if they intended on getting the vaccine as soon as possible; (2) “hesitant” if they intended to wait to see how it affects others in the community before getting it or not intending to get it soon, but might sometime in the future; and (3) “do not intend to get vaccinated” if they did not intend on ever getting the vaccine.

*Cognitive predictors: Trust in government.* Participants were asked to rate whether they agreed that “*Most of the time, we can trust people in the provincial government to do the right thing*” using a Likert scale ranging from 1 = do not agree to 5 = agree completely (Franzen & Vogl, 2013).

*COVID-19 conspiracy theory beliefs.* Conspiracy theory beliefs specific to COVID-19 were assessed through four statements adapted from Freeman et al. (2022); “*The government is misleading the public about the cause of the Coronavirus*”, “*The spread of the Coronavirus is a deliberate attempt by a group of powerful people to gain control*”, “*Coronavirus is a bioweapon developed by China to destroy the West”*, and “*The mainstream media is deliberately feeding us misinformation about the Coronavirus and lockdown*”. Participants indicated the extent to which they agreed with each statement using a scale from 1 = do not agree to 5 = agree completely.

*General attitudes towards vaccines.* General attitudes towards vaccines were assessed through the Vaccination Attitudes Examination (VAX) Scale (Martin & Petrie, 2017). Sample items include “ *I feel safe after being vaccinated*” and “*Natural immunity lasts longer than a vaccination*”. Using a Likert scale from 1 = strongly disagree to 7 = strongly agree, participants indicated whether they agreed with each of the 12 items, with higher scores indicating more negative attitudes towards vaccines (McDonald's Ω at T1 = 0.92, and at T2 = 0.93).

*Vaccine factors.* Participants were presented with eight statements from the C4R Questionnaire (C4R Investigators, 2020) and asked, “For each option, would you agree or disagree that this factor affects your opinion about a vaccine?”. Participants were asked to answer using a Likert scale from 1 = strongly disagree to 7 = strongly agree. The statements are as follows: “*The current politics*”, “*The rushed/fast-tracked research and development timeline*”, “*The frequently changing science of COVID-19*”, “*Actions and opinions of my friends and family regarding the vaccine*”, “*My trust in scientists*”, “*My own reading and research on COVID-19 vaccines*”, “*The country in which a vaccine is manufactured*”, and “*The potential cost of a coronavirus (COVID-19) vaccine*”.

*Socio-emotional predictors: Psychological distress.* Psychological distress was measured using the Hopkins Symptom Checklist-25 (HSCL-25), which has good psychometric properties (McDonald's Ω at T1 and T2 = 0.96) (Derogatis et al., 1974; Mollica et al., 1992). Items were scored on a 4-point Likert scale based on how much discomfort that problem has caused them during the past 7 days from 1 (not at all) to 4 (extremely). The total score was calculated as the mean of all items. Sample items include: “*Suddenly scared for no reason*”and “*Feeling sad*”.

*Social support.* The Multidimensional Scale of Perceived Social Support (MSPSS) from extended family and friends was assessed using an eight-item scale (McDonald's Ω at T1 and T2 = 0.92) (Canty-Mitchell & Zimet, 2000). Sample items include “*My family really tries to help me*” as well as “*I can talk about my problems with my friends*” and are scored on a 7-point Likert scale from 1 (strongly disagree) to 7 (strongly agree), with higher scores indicating more perceived social support.

*Covariates: Socio-demographic characteristics.* Participants were asked to provide information including province of residence, age, gender (man, woman, other), marital status (never married, divorced/separated/widowed, married/living together as a couple), household income (≤$19,999, $20,000–$39,999, $40,000–$59,9999, $60,000–$79,9999, $80,000–$99,9999, ≥$100,000), education level (none/less than high school, high school graduate, any year of apprenticeship or technical institute or trade or vocational school, any year of College or CEGEP or other non-university certificate or diploma, any year of University certificate or diploma or degree), religion (no religion, religion), and immigration generation (first, second, third or more).

### Data analysis

All statistical analyses were conducted using R version 4.2.1 (R Core Team, 2023) and RStudio Version 2022.7.2.576 (RStudio Team, 2022). We calculated descriptive statistics (mean and standard deviation for continuous variables and counts and proportions for categorical variables) at T1 and T2 for participants responding to the survey at both time points. Results of the attrition analysis are reported in the Supplemental Material. Missing values were imputed with the random forest technique with the *missForest* package (Stekhoven & Bühlmann, 2012). This technique of multiple imputation is particularly suited for large datasets including both numeric and categorical variables as it does not require a model specification by single variables and allows for the inclusion of complex interactions between categorical and numeric variables (Stekhoven & Bühlmann, 2012). The process terminated after six iterations with final out-of-box imputation error estimates NRMSE = 0.007 (normalized root mean squared error, for continuous variables), and PCF = 0.230 (proportion of falsely classified entries, for categorical variables). Sensitivity analyses indicated that imputed missing values maintained integrity of results.

To account for the violation of independency in our data due to the longitudinal design, we used Generalized Estimating Equation (GEE) modeling with the *multgee* package (Touloumis, 2014). This package allows for estimating associations between the correlated measurements of the multinomial vaccine hesitancy outcome variable and predictors at the population level, while controlling for intra-individual correlation. All models were adjusted for time-varying and static socio-demographic variables (i.e., gender, age, province, generation, highest education grade, income, marital status, religion). First, we investigated the effect of time on the vaccine hesitancy outcome as well as on our predictors. Second, we investigated the main effect of each predictor on vaccine hesitancy, in separate models. Last, we investigated the independent associations of the predictors that were significantly associated with our outcome in step 2 simultaneously in one model.

## Results

The number of participants who reported vaccine hesitancy decreased over time, with 7.6% of participants reporting being “hesitant” or “not intending to get vaccinated” at T2 compared to 17.9% at T1 (Table 1). The corresponding odds ratios (ORs) for status “hesitant” and “do not intend to get vaccinated”, with “not hesitant” as reference category, at T2 compared to T1 (controlling for socio-demographic variables), were also statistically significant (*p *< .001; Table 2).

**Table 2.** Generalized estimating equation models for the effect of time on vaccine hesitancy and potential predictors of vaccine hesitancy (*N* = 2,695).

	Time point (T2 vs T1)		
Outcome	β	95% CI	*p*-value
Psychological distress	0.06	0.03, 0.09	<.001
Social support (total)	−0.39	−0.93, 0.15	.153
Trust in government	0.04	−0.01, 0.10	.119
COVID-19 conspiracy theories*			
*1*	−0.06	−0.12, 0.01	.076
*2*	−0.01	−0.07, 0.04	.695
*3*	0.00	−0.06, 0.05	.909
*4*	−0.05	−0.11, 0.02	.165
Vaccine attitudes (VAX)	−0.02	−0.09, 0.04	.467
Vaccine factors**			
*1*	0.00	−0.08, 0.09	.941
*2*	−0.27	−0.36, −0.18	<.001
*3*	−0.14	−0.22, −0.06	.001
*4*	−0.13	−0.21, −0.04	.005
*5*	−0.01	−0.09, 0.07	.833
*6*	−0.15	−0.22, −0.07	<.001
*7*	0.02	−0.07, 0.11	.681
*8*	0.01	−0.08, 0.10	.867
Vaccine hesitancy	*OR*	95% CI	*p*-value
Hesitant vs Not hesitant	0.24	0.20, 0.29	<.001
Do not intend to get vaccinated vs Not hesitant	0.64	0.54, 0.75	<.001

*Note.* * = COVID-19 conspiracy theories 1: “The government is misleading the public about the cause of the Coronavirus”, 2: “The spread of the Coronavirus is a deliberate attempt by a group of powerful people to gain control”, 3: “Coronavirus is a bioweapon developed by China to destroy the West”, and 4: “The mainstream media is deliberately feeding us misinformation about the Coronavirus and lockdown”; ** = Vaccine factors 1: “The current politics”, 2: “The rushed/fast-tracked research and development timeline”, 3: “The frequently changing science of COVID-19”, 4: “Actions and opinions of my friends and family regarding the vaccine”, 5: “My trust in scientists”, 6: “My own reading and research on COVID-19 vaccines”, 7: “The country in which a vaccine is manufactured”, 8: “The potential cost of a coronavirus (COVID-19) vaccine”. CI = confidence interval; *OR* = odds ratio. All models were adjusted for age, gender, province, immigrant generation, religiosity, marital status, household income and education.

With regard to changes in cognitive factors over time, the influence in decision-making reported for COVID-19 vaccine factors related to the rushed/fast-tracked development of the vaccines, the frequently changing science of COVID-19, the actions and opinions of friends and family regarding the vaccine and participants’ own reading around vaccines decreased from T1 to T2. Trust in government, endorsement of COVID-19 conspiracy beliefs, and vaccine attitudes did not change significantly over time (Table 2). With regard to socio-emotional factors, psychological distress increased from T1 to T2, whereas reported social support did not change significantly over time (Table 2).

Results from separate multinomial GEE models indicated that all cognitive factors except for the conspiracy beliefs around the coronavirus being a bioweapon developed by China to destroy the West and the COVID-19-related factor on the potential cost of a COVID-19 vaccine were statistically significant predictors of vaccine hesitancy (similarly for “hesitant” and “do not intend to get vaccinated” participants) (Table 3). With regard to socio-emotional factors, higher psychological distress was a statistically significant predictor of a lower likelihood of “do not intend to get vaccinated” compared to “not hesitant” participants, with no statistically significant differences between “hesitant” and “not hesitant” participants. Higher social support was a statistically significant predictor of a lower likelihood of being “hesitant” compared to “not hesitant” (Table 3).

**Table 3.** Multinomial generalized estimating equation models for the association between potential predictors with vaccine hesitancy (“not hesitant” as reference cateogory)(*N* = 2,695).

		Hesitant			Do not intend to get vaccinated		
#	Predictors	*OR*	95% CI	*p*-value	*OR*	95% CI	*p*-value
1	Psychological distress	1.14	0.95–1.37	.166	0.62	0.46–0.82	.001
2	Social support (total)	0.98	0.98–0.99	.003	0.99	0.98–1.01	.369
3	Trust in government	0.78	0.70–0.87	<.001	0.51	0.43–0.61	<.001
4	COVID-19 conspiracy theories*						
	*1*	1.15	1.01–1.31	.031	1.31	1.07–1.61	.008
	*2*	1.30	1.12–1.52	.001	1.63	1.34–1.98	<.001
	*3*	0.93	0.81–1.07	.304	0.87	0.73–1.04	.133
	*4*	1.23	1.10–1.39	<.001	1.62	1.36–1.94	<.001
5	Vaccine attitudes (VAX)	2.63	2.40–2.89	<.001	5.44	4.51–6.57	<.001
6	Vaccine factors**						
	*1*	1.09	1.01–1.17	.026	1.24	1.14–1.35	<.001
	*2*	1.46	1.32–1.61	<.001	1.35	1.19–1.53	<.001
	*3*	1.28	1.15–1.41	<.001	1.20	1.07–1.33	.001
	*4*	0.93	0.87–1.00	.054	0.85	0.77–0.94	.002
	*5*	0.69	0.64–0.74	<.001	0.75	0.68–0.82	<.001
	*6*	0.91	0.84–0.99	.031	1.20	1.08–1.34	.001
	*7*	0.91	0.85–0.98	.016	0.83	0.77–0.90	<.001
	*8*	0.97	0.90–1.04	.379	0.95	0.87–1.03	.217

*Note.* * = COVID-19 conspiracy theories 1: “The government is misleading the public about the cause of the Coronavirus”, 2: “The spread of the Coronavirus is a deliberate attempt by a group of powerful people to gain control”, 3: “Coronavirus is a bioweapon developed by China to destroy the West”, and 4: “The mainstream media is deliberately feeding us misinformation about the Coronavirus and lockdown”; ** = Vaccine factors 1: “The current politics”, 2: “The rushed/fast-tracked research and development timeline”, 3: “The frequently changing science of COVID-19”, 4: “Actions and opinions of my friends and family regarding the vaccine”, 5: “My trust in scientists”, 6: “My own reading and research on COVID-19 vaccines”, 7: “The country in which a vaccine is manufactured”, 8: “The potential cost of a coronavirus (COVID-19) vaccine”. CI = confidence interval; *OR* = odds ratio. All models were adjusted for time point, age, gender, province, immigrant generation, religiosity, marital status, household income and education.

Table 4 reports results of the independent associations between each predictor and vaccine hesitancy by including all predictors that were significantly associated with our outcome in prior analyses in one model, while adjusting for socio-demographic variables and time. Results indicated that higher trust in government was associated with a lower likelihood of vaccine hesitancy (for both “hesitant” and “do not intend to get vaccinated” participants). Negative attitudes towards vaccines and conspiracy beliefs around the spread of COVID-19 being a deliberate attempt by a group of powerful people to gain control were both significant predictors of vaccine hesitancy (both hesitant statuses). The reported influence of the frequently changing science of COVID-19 remained statistically associated with a higher likelihood of vaccine hesitancy (both statuses), whereas COVID-19-related factors around actions and opinions of friends and family regarding the vaccine as well as related to the country in which a vaccine was manufactured remained statistically significant predictors of a lower likelihood of vaccine hesitancy (both hesitant statuses). The factor related to the rushed/fast-tracked research and development timeline of the COVID-19 vaccine was a statistically significant predictor of a higher likelihood of being “hesitant” compared to “not hesitant”, whereas factors around trust in scientists and participants’ own reading and research on COVID-19 vaccines were significant predictors of a lower likelihood of being “hesitant” compared to “not hesitant”. Endorsement of conspiracy beliefs around the mainstream media deliberately feeding misinformation about the Coronavirus and lockdown as well as beliefs around current politics remained significant predictors of a higher likelihood of participants who “do not intend to get vaccinated” compared to “not hesitant” participants.

**Table 4.** Multinomial generalized estimating equation model estimating the independent associations between potential predictors and vaccine hesitancy (“not hesitant” as reference category)(*N* = 2,695).

	Hesitant			Do not intend to get vaccinated		
Predictors	*OR*	95% CI	*p*-value	*OR*	95% CI	*p*-value
Psychological distress	0.83	0.67–1.02	.072	0.35	0.24–0.52	<.001
Social support (total)	0.99	0.98–1.00	.036	0.98	0.96–1.00	.033
Trust in government	0.88	0.78–1.00	.045	0.65	0.50–0.84	.001
COVID-19 conspiracy theories*						
*1*	0.95	0.83–1.09	.489	1.03	0.82–1.30	.803
*2*	1.17	1.02–1.33	.022	1.56	1.27–1.91	<.001
*4*	1.06	0.93–1.21	.362	1.25	1.01–1.55	.043
Vaccine attitudes (VAX)	2.18	1.90–2.49	<.001	3.41	2.66–4.36	<.001
Vaccine factors**						
*1*	1.05	0.97–1.14	.255	1.28	1.14–1.43	<.001
*2*	1.30	1.18–1.44	<.001	1.13	0.94–1.36	.179
*3*	1.26	1.13–1.39	<.001	1.26	1.05–1.50	.013
*4*	0.88	0.81–0.95	.001	0.82	0.72–0.94	.004
*5*	0.79	0.72–0.86	<.001	0.90	0.77–1.05	.196
*6*	0.86	0.79–0.94	.001	0.94	0.81–1.09	.399
*7*	0.84	0.78–0.91	<.001	0.72	0.64–0.81	<.001

*Note.* * = COVID-19 conspiracy theories 1: “The government is misleading the public about the cause of the Coronavirus”, 2: “The spread of the Coronavirus is a deliberate attempt by a group of powerful people to gain control”, 3: “Coronavirus is a bioweapon developed by China to destroy the West”, and 4: “The mainstream media is deliberately feeding us misinformation about the Coronavirus and lockdown”; ** = Vaccine factors 1: “The current politics”, 2: “The rushed/fast-tracked research and development timeline”, 3: “The frequently changing science of COVID-19”, 4: “Actions and opinions of my friends and family regarding the vaccine”, 5: “My trust in scientists”, 6: “My own reading and research on COVID-19 vaccines”, 7: “The country in which a vaccine is manufactured”. CI = confidence interval; OR = odds ratio. All models were adjusted for time point, age, gender, province, immigrant generation, religiosity, marital status, household income and education.

Lower psychological distress remained a statistically significant predictor of “do not intend to get vaccinated” compared to “not hesitant” participants, with no statistically significant differences between “hesitant” and “not hesitant” participants. Higher social support was a statistically significant predictor of a lower likelihood af being both “hesitant” and “do not intend to get vaccinated” compared to “not hesitant” participants.

## Discussion

Findings indicated a decrease in vaccine hesitancy from June to November 2021, consistent with the observed increase in the prevalence of COVID-19 vaccination rates across provinces in the Canadian context (PHAC, 2022). The introduction of vaccine mandates in September played a role, although this policy did not manage to motivate the full population to get vaccinated. This confirms preliminary data showing that coercive vaccination is unlikely to be effective in reaching the significant segment of the population which holds alternative beliefs regarding COVID-19 and vaccines (Lord, 2021). We found that, over time, scores on trust in government and vaccine attitudes in general remained stable, despite the increase in vaccination intake. We found no change in conspiracy beliefs over time, which contradicts common public discourses around the increase of such beliefs in recent times and confirms empirical findings on the overall stability of such beliefs in the general population in the past decade (Uscinski et al., 2022). Once established, cognitive attitudes and conspiracy beliefs may not be changed by simply focusing on rational arguments (Jolley et al., 2018). However, some changes in the importance of specific cognitive factors in influencing one's decision around vaccination were found. Specifically, our results pointed to an overall decrease of the influence of worries around scientific research about COVID-19 vaccines. This may be attributed to growing empirical evidence on the effectiveness of COVID-19 vaccines over time which may have mitigated anxiety about the vaccine. The reported influence of friends and family on vaccination intentions also decreased over time, suggesting that one's social network may influence the decision-making process more at the beginning of vaccination campaigns (Salali & Uysal, 2023). With regard to socio-emotional factors, we found a significant increase in psychological distress over a 6-month period. This finding confirms the literature on COVID-19 fatigue, which underlines the negative impact of the pandemic on mental health, providing support to mounting evidence on the long-term consequences of the pandemic on socio-emotional adjustment among young people (Bardosh et al., 2022; Cooper, 2019; Pearce & Cooper, 2021).

Notably, the overall reduction in vaccine hesitancy in our sample was associated with an increase in psychological distress. Specifically, we found that participants who were “not hesitant” at T2 were more likely to report higher psychological distress compared to participants who “did not intend to get vaccinated”. Some studies conducted before the pandemic suggest that people who are more depressed or anxious are overall more likely to get vaccinated (Chan et al., 2015; Koltai et al., 2022; Lawrence et al., 2020; Mohammed-Roberts et al., 2020; Nguyen, 2021); this interpretation cannot be fully excluded without more rigorous longitudinal studies monitoring both psychological distress and vaccine hesitancy across multiple time points (Perez-Arce et al., 2021). Nonetheless, our finding may be interpreted in light of the limited personal benefits experienced with vaccination for younger participants (Chaudhuri et al., 2022). It is possible that the COVID-19 fatigue in the long-term, together with vaccine mandates that were not necessarily accompanied by a release of social distancing measures nor by an outstanding reduction of infections, may have further reduced the perception of the benefits of vaccination among young people and increased cognitive dissonance among “not hesitant” participants, contributing to feelings of impotence and thus negatively impacting their mental health (Bardosh et al., 2022; Li et al., 2022; Pearce & Cooper, 2021). In contrast, the lower psychological distress reported by participants who did not intend to get vaccinated raises the hypothesis that defying vaccine mandates may have allowed young people to actively express their discontent, thus representing a source of empowerment and personal agency which may have contributed to the creation of alternative support networks, positively impacting their mental health. Of note, during these six months, vaccine mandates were introduced and no-vax movements gained in popularity and visibility, influencing to some extent public health decisions. For instance, in November 2021, the Quebec government backed down on mandatory vaccination for health employees due to protests (Lowrie, 2021). It is thus possible to think that the protesters felt they finally had a voice, and that their predicament of refusing vaccination gained in legitimacy.

Our findings indicate that “hesitant” participants as well as participants who “did not intend to get vaccinated” reported lower social support compared to “not hesitant” participants, suggesting that isolation and lack of social support are determinants of vaccine hesitancy (Moscardino et al., 2022). In light of evidence that vaccine hesitancy and the polarization around COVID-19 vaccines have contributed to interpersonal conflicts and the erosion of existing social support networks (Heyerdahl et al., 2023; Santavicca, Vanier-Clément, & Rosusseau, 2022), it is important that public health initiatives and messages adopt a non-stigmatizing and inclusive approach in vaccination campaigns to enable reducing social and interpersonal tensions in the population and protect individuals from isolation.

Regarding cognitive factors, as expected, lower trust in government and negative attitudes towards vaccines were both significantly associated with vaccine hesitancy, confirming prior literature (Moscardino et al., 2022; Santavicca, Ngov, et al., 2022; Van Oost et al., 2022). Only some conspiracy beliefs were significantly associated with the intention of not getting vaccinated against COVID-19, supporting preliminary findings on the differential impact of specific misbeliefs on vaccine hesitancy (Santavicca, Ngov, et al., 2022). Differences were found among the independent predictors of “hesitant” and “did not intend to get vaccinated” participants, suggesting that public health and media communication strategies should acknowledge the nuances associated with vaccine hesitancy and different conspiracy beliefs and address these differences in policies and messages (Santavicca, Ngov, et al., 2022). Similarly, the COVID-19 factors that were reported to influence one's decision-making around vaccination also varied between “hesitant” and “did not intend to get vaccinated” participants. Aspects related to the science behind the COVID-19 vaccine and participants’ own reading and research around COVID-19 vaccines were more consistently predictors of participants being “hesitant” compared to “not hesitant”; however, they did not seem to play a significant role among participants who “did not intend to get vaccinated”. This may suggest that active efforts to inform oneself as to be able to make an informed decision may be a protective behaviour to be supported as an empowering way to promote vaccination uptake among undecided individuals.

The fact that both cognitive and socio-emotional predictors showed a significant independent association with vaccine hesitancy further supports the importance of considering dissent as the result of a complex decision-making process that involves both socio-emotional and cognitive components (Dubé & Macdonald, 2022; Hicks & Lloyd, 2022). Both individual and colletive benefits and costs of complying with public health guidelines need to be carefully considered and balanced, paying attention to the need of personal agency and empowerment among young adults in times of uncertainty. This recommendation confirms prior findings showing that people who had fewer negative experiences during the pandemic (i.e., who perceived more benefits than costs related to public health measures) were more likely to follow public health guidelines (Lyu et al., 2022). Public health vaccination campaigns and preventive efforts that jointly target cognitive and socio-emotional factors should be privileged and prioritize the promotion of mental health and empowerment among young people (Lundström, 2022). Media and information literacy as well as trust in governments have been highly targeted in vaccination campaigns (Hicks & Lloyd, 2022). Although such efforts may be effective and needed to some extent, our results seem to suggest that other cognitive aspects related to empowerment (i.e., active research online and the scientific process of vaccine development) may be equally important to address. Beyond these generally accepted considerations, the present paradoxical findings about psychological distress and dissent suggest that authoritarian approaches during crisis times may have unintended consequences and elicit resistance strategies which may play a protective role for some individuals. This supports the idea that democratic communications and transparency may in the long run promote a more trusting relationship towards institutions and governments than coercion (Bardosh et al., 2022; Cairney & Wellstead, 2020). However, further studies are needed to confirm this preliminary interpretation and to unpack the mechanisms at play.

Results should be interpreted in light of several limitations. First, vaccine hesitancy was used as a proxy for dissent, which is a complex construct that englobes many nuances that we could not capture in our study. Future studies should assess dissent with more fine-grained measures. Mixed-method studies that conduct a more in-depth qualitative exploration of meaning of dissent among young people and empirically investigate empowerment and cognitive dissonance in times of social polarization are warranted to further validate our interpretations. Second, our study relied exclusively on self-reported measures and results are therefore subject to social desirability bias. In addition, social media use was not considered in the present study and should be addressed in future surveys given the important role it plays among young people as well as in the vaccine hesitancy literature. Third, we relied on a convenience sample based on an initial limited respose rate at T1 (10%). Furthermore, the attrition rate was high (46%), compromising the generalizability of findings. Nonetheless, the sample was large and our models controlled for socio-demographic variables that were significantly associated with drop-out rates. Last, results cannot be generalized to other Canadian provinces or countries, nor to other phases of the pandemic given the specificities of the Canadian context at the time of the study.

Despite these limitations, our study raises important questions around the role of cognitive and socio-emotional predictors of dissent during an extended period of social unrest, i.e., a long pandemic, and underlines the need to better understand the potential role that expression of dissent can play in decreasing psychological distress and increasing agency among young adults during a global health crisis (Lundström, 2022).

## Supplemental Material

sj-docx-1-tps-10.1177_13634615241296293 - Supplemental material for The protective power of dissent? A longitudinal study on cognitive and socio-emotional determinants of COVID-19 vaccine hesitancy among young people in Canada Supplemental material, sj-docx-1-tps-10.1177_13634615241296293 for The protective power of dissent? A longitudinal study on cognitive and socio-emotional determinants of COVID-19 vaccine hesitancy among young people in Canada by Diana Miconi, Anna Levinsson, Mohammed Abdullah Heel Kafi, Cindy Ngov, Tara Santavicca, and Cécile Rousseau in Transcultural Psychiatry
