# Supplementary material for: The protective power of dissent? A longitudinal study on cognitive and socio-emotional determinants of COVID-19 vaccine hesitancy among young people in Canada
Source: Transcult Psychiatry. 2024 Nov 18;61(5):734–48. doi: 10.1177/13634615241296293 (PMC11629591; doi:10.1177/13634615241296293)
Supplement: sj-docx-1-tps-10.1177_13634615241296293 - Supplemental material for The protective power of dissent? A longitudinal study on cognitive and socio-emotional determinants of COVID-19 vaccine hesitancy among young people in Canada [file sj-docx-1-tps-10.1177_13634615241296293.docx]

Supplemental materials

Table 1. Comparison of T1 characteristics for participant groups

|  | 1st wave only (N=2312) | 1st and 2nd wave (N=2695) | p-value |
| --- | --- | --- | --- |
| **Age (years)** |  |  |  |
| Mean (SD) | 27.8 (5.61) | 29.6 (5.51) | <0.001 |
| **Gender (n [%])** |  |  |  |
| Woman | 1354 (58.6%) | 1605 (59.6%) | 0.593 |
| Man | 940 (40.7%) | 1073 (39.8%) |  |
| Transgender or gender-diverse | 12 (0.5%) | 10 (0.4%) |  |
| Missing | 6 (0.3%) | 7 (0.3%) |  |
| **Province (n [%])** |  |  |  |
| Alberta | 533 (23.1%) | 599 (22.2%) | 0.623 |
| Ontario | 938 (40.6%) | 1128 (41.9%) |  |
| Quebec | 841 (36.4%) | 968 (35.9%) |  |
| **Immigrant generation (n [%])** |  |  |  |
| First | 427 (18.5%) | 492 (18.3%) | 0.002 |
| Second | 446 (19.3%) | 633 (23.5%) |  |
| Third or more | 1417 (61.3%) | 1559 (57.8%) |  |
| Missing | 22 (1.0%) | 11 (0.4%) |  |
| **Religiosity (n [%])** |  |  |  |
| No religion | 1100 (47.6%) | 1338 (49.6%) | 0.201 |
| Religious | 1099 (47.5%) | 1239 (46.0%) |  |
| Missing | 113 (4.9%) | 118 (4.4%) |  |
| **Marital status (n [%])** |  |  |  |
| Never married | 1253 (54.2%) | 1340 (49.7%) | 0.001 |
| Divorced/separated/widowed | 61 (2.6%) | 67 (2.5%) |  |
| Married/common law/living together | 962 (41.6%) | 1270 (47.1%) |  |
| Missing | 36 (1.6%) | 18 (0.7%) |  |
| **Education (n [%])** |  |  |  |
| None/Less than high school | 58 (2.5%) | 45 (1.7%) | <0.001 |
| High school graduate | 482 (20.8%) | 399 (14.8%) |  |
| Apprenticeship, technical institute, trade or vocational school (any year) | 145 (6.3%) | 161 (6.0%) |  |
| College, CEGEP or other non-university certificate or diploma (any year) | 531 (23.0%) | 573 (21.3%) |  |
| University certificate, diploma or degree (any year) | 1068 (46.2%) | 1504 (55.8%) |  |
| Missing | 28 (1.2%) | 13 (0.5%) |  |
| **Income (n [%])** |  |  |  |
| $19,999 or less | 221 (9.6%) | 144 (5.3%) | <0.001 |
| Between $20,000 and $39,999 | 313 (13.5%) | 335 (12.4%) |  |
| Between $40,000 and $59,999 | 367 (15.9%) | 380 (14.1%) |  |
| Between $60,000 and $79,999 | 363 (15.7%) | 401 (14.9%) |  |
| Between $80,000 and $99,999 | 285 (12.3%) | 416 (15.4%) |  |
| $100,000 or more | 538 (23.3%) | 741 (27.5%) |  |
| Missing | 225 (9.7%) | 278 (10.3%) |  |
| **Psychological distress** |  |  |  |
| Mean (SD) | 1.77 (0.604) | 1.69 (0.567) | <0.001 |
| Missing | 144 (6.2%) | 156 (5.8%) |  |
| **Trust in government** |  |  |  |
| Mean (SD) | 2.67 (1.15) | 2.65 (1.10) | 0.610 |
| Missing | 46 (2.0%) | 59 (2.2%) |  |
| **COVID-19 conspiracy theory 1** |  |  |  |
| Mean (SD) | 2.04 (1.22) | 1.99 (1.21) | 0.196 |
| Missing | 112 (4.8%) | 137 (5.1%) |  |
| **COVID-19 conspiracy theory 2** |  |  |  |
| Mean (SD) | 1.72 (1.14) | 1.66 (1.11) | 0.079 |
| Missing | 118 (5.1%) | 132 (4.9%) |  |
| **COVID-19 conspiracy theory 3** |  |  |  |
| Mean (SD) | 1.68 (1.13) | 1.61 (1.05) | 0.030 |
| Missing | 158 (6.8%) | 199 (7.4%) |  |
| **COVID-19 conspiracy theory 4** |  |  |  |
| Mean (SD) | 2.26 (1.30) | 2.12 (1.26) | <0.001 |
| Missing | 93 (4.0%) | 106 (3.9%) |  |
| **Social support (total)** |  |  |  |
| Mean (SD) | 42.6 (10.4) | 42.0 (10.6) | 0.043 |
| Missing | 68 (2.9%) | 100 (3.7%) |  |
| **Vaccine attitudes (VAX)** |  |  |  |
| Mean (SD) | 3.33 (1.27) | 3.26 (1.25) | 0.103 |
| Missing | 266 (11.5%) | 314 (11.7%) |  |
| **Vaccination hesitancy (n [%])** |  |  |  |
| Do not intend to get vaccinated | 118 (5.1%) | 148 (5.5%) | 0.785 |
| Hesitant | 276 (11.9%) | 333 (12.4%) |  |
| Not hesitant | 1863 (80.6%) | 2167 (80.4%) |  |
| Missing | 55 (2.4%) | 47 (1.7%) |  |
| **Vaccine factor 1** |  |  |  |
| Mean (SD) | 3.93 (1.75) | 3.95 (1.64) | 0.581 |
| Missing | 65 (2.8%) | 77 (2.9%) |  |
| **Vaccine factor 2** |  |  |  |
| Mean (SD) | 4.37 (1.75) | 4.38 (1.75) | 0.787 |
| Missing | 57 (2.5%) | 67 (2.5%) |  |
| **Vaccine factor 3** |  |  |  |
| Mean (SD) | 4.67 (1.61) | 4.68 (1.57) | 0.811 |
| Missing | 53 (2.3%) | 73 (2.7%) |  |
| **Vaccine factor 4** |  |  |  |
| Mean (SD) | 4.12 (1.75) | 4.10 (1.70) | 0.624 |
| Missing | 43 (1.9%) | 54 (2.0%) |  |
| **Vaccine factor 5** |  |  |  |
| Mean (SD) | 5.35 (1.55) | 5.38 (1.49) | 0.539 |
| Missing | 38 (1.6%) | 51 (1.9%) |  |
| **Vaccine factor 6** |  |  |  |
| Mean (SD) | 4.87 (1.59) | 4.85 (1.53) | 0.645 |
| Missing | 61 (2.6%) | 74 (2.7%) |  |
| **Vaccine factor 7** |  |  |  |
| Mean (SD) | 4.00 (1.75) | 4.10 (1.70) | 0.046 |
| Missing | 40 (1.7%) | 72 (2.7%) |  |
| **Vaccine factor 8** |  |  |  |
| Mean (SD) | 3.57 (1.73) | 3.62 (1.73) | 0.319 |
| Missing | 69 (3.0%) | 71 (2.6%) |  |
| Note. SD: standard deviation | | | |

Table 2. Comparison of characteristics at T1 between the vaccine hesitancy groups at T1

|  | Do not intend to get vaccinated (N=266) | Hesitant (N=609) | Not hesitant (N=4030) | p-value |
| --- | --- | --- | --- | --- |
| **Age (years)** |  |  |  |  |
| Mean (SD) | 29.3 (5.53) | 29.0 (5.45) | 28.7 (5.65) | 0.225 |
| **Gender** |  |  |  |  |
| Woman | 162 (60.9%) | 403 (66.2%) | 2333 (57.9%) | 0.003 |
| Man | 99 (37.2%) | 202 (33.2%) | 1671 (41.5%) |  |
| Transgender or gender-diverse | 1 (0.4%) | 3 (0.5%) | 18 (0.4%) |  |
| Missing | 4 (1.5%) | 1 (0.2%) | 8 (0.2%) |  |
| **Province** |  |  |  |  |
| Alberta | 75 (28.2%) | 164 (26.9%) | 865 (21.5%) | <0.001 |
| Ontario | 88 (33.1%) | 275 (45.2%) | 1658 (41.1%) |  |
| Quebec | 103 (38.7%) | 170 (27.9%) | 1507 (37.4%) |  |
| **Immigrant generation** |  |  |  |  |
| First | 31 (11.7%) | 125 (20.5%) | 741 (18.4%) | 0.005 |
| Second | 51 (19.2%) | 144 (23.6%) | 864 (21.4%) |  |
| Third or more | 182 (68.4%) | 339 (55.7%) | 2401 (59.6%) |  |
| Missing | 2 (0.8%) | 1 (0.2%) | 24 (0.6%) |  |
| **Religiosity** |  |  |  |  |
| No religion | 121 (45.5%) | 273 (44.8%) | 2011 (49.9%) | 0.038 |
| Religious | 132 (49.6%) | 309 (50.7%) | 1853 (46.0%) |  |
| Missing | 13 (4.9%) | 27 (4.4%) | 166 (4.1%) |  |
| **Marital status** |  |  |  |  |
| Never married | 136 (51.1%) | 285 (46.8%) | 2127 (52.8%) | 0.066 |
| Divorced/separated/widowed | 6 (2.3%) | 20 (3.3%) | 97 (2.4%) |  |
| Married/common law/living together | 119 (44.7%) | 300 (49.3%) | 1767 (43.8%) |  |
| Missing | 5 (1.9%) | 4 (0.7%) | 39 (1.0%) |  |
| **Education** |  |  |  |  |
| None/Less than high school | 15 (5.6%) | 29 (4.8%) | 54 (1.3%) | <0.001 |
| High school graduate | 82 (30.8%) | 162 (26.6%) | 614 (15.2%) |  |
| Apprenticeship, technical institute, trade or vocational school (any year) | 33 (12.4%) | 57 (9.4%) | 208 (5.2%) |  |
| College, CEGEP or other non-university certificate or diploma (any year) | 78 (29.3%) | 140 (23.0%) | 865 (21.5%) |  |
| University certificate, diploma or degree (any year) | 57 (21.4%) | 218 (35.8%) | 2255 (56.0%) |  |
| Missing | 1 (0.4%) | 3 (0.5%) | 34 (0.8%) |  |
| **Income** |  |  |  |  |
| $19,999 or less | 39 (14.7%) | 74 (12.2%) | 244 (6.1%) | <0.001 |
| Between $20,000 and $39,999 | 43 (16.2%) | 107 (17.6%) | 479 (11.9%) |  |
| Between $40,000 and $59,999 | 35 (13.2%) | 105 (17.2%) | 588 (14.6%) |  |
| Between $60,000 and $79,999 | 42 (15.8%) | 95 (15.6%) | 618 (15.3%) |  |
| Between $80,000 and $99,999 | 34 (12.8%) | 66 (10.8%) | 589 (14.6%) |  |
| $100,000 or more | 44 (16.5%) | 105 (17.2%) | 1120 (27.8%) |  |
| Missing | 29 (10.9%) | 57 (9.4%) | 392 (9.7%) |  |
| **Psychological distress** |  |  |  |  |
| Mean (SD) | 1.63 (0.566) | 1.82 (0.646) | 1.72 (0.577) | <0.001 |
| Missing | 20 (7.5%) | 41 (6.7%) | 214 (5.3%) |  |
| **Trust in government** |  |  |  |  |
| Mean (SD) | 1.81 (1.02) | 2.29 (0.985) | 2.78 (1.11) | <0.001 |
| Missing | 6 (2.3%) | 17 (2.8%) | 70 (1.7%) |  |
| **COVID-19 conspiracy theory 1** |  |  |  |  |
| Mean (SD) | 3.80 (1.32) | 2.70 (1.28) | 1.78 (1.04) | <0.001 |
| Missing | 18 (6.8%) | 39 (6.4%) | 161 (4.0%) |  |
| **COVID-19 conspiracy theory 2** |  |  |  |  |
| Mean (SD) | 3.35 (1.47) | 2.27 (1.29) | 1.48 (0.928) | <0.001 |
| Missing | 20 (7.5%) | 46 (7.6%) | 156 (3.9%) |  |
| **COVID-19 conspiracy theory 3** |  |  |  |  |
| Mean (SD) | 2.72 (1.50) | 1.98 (1.19) | 1.52 (0.986) | <0.001 |
| Missing | 34 (12.8%) | 67 (11.0%) | 224 (5.6%) |  |
| **COVID-19 conspiracy theory 4** |  |  |  |  |
| Mean (SD) | 3.98 (1.23) | 2.84 (1.27) | 1.96 (1.15) | <0.001 |
| Missing | 15 (5.6%) | 35 (5.7%) | 122 (3.0%) |  |
| **Social support (total)** |  |  |  |  |
| Mean (SD) | 39.8 (12.2) | 40.2 (11.2) | 42.8 (10.2) | <0.001 |
| Missing | 16 (6.0%) | 29 (4.8%) | 109 (2.7%) |  |
| **Vaccine attitudes (VAX)** |  |  |  |  |
| Mean (SD) | 5.62 (1.07) | 4.39 (0.872) | 2.98 (1.06) | <0.001 |
| Missing | 43 (16.2%) | 103 (16.9%) | 396 (9.8%) |  |
| **Vaccine factor 1** |  |  |  |  |
| Mean (SD) | 4.72 (2.07) | 4.38 (1.67) | 3.82 (1.65) | <0.001 |
| Missing | 16 (6.0%) | 18 (3.0%) | 93 (2.3%) |  |
| **Vaccine factor 2** |  |  |  |  |
| Mean (SD) | 5.44 (2.05) | 5.35 (1.64) | 4.15 (1.67) | <0.001 |
| Missing | 11 (4.1%) | 11 (1.8%) | 89 (2.2%) |  |
| **Vaccine factor 3** |  |  |  |  |
| Mean (SD) | 5.33 (1.85) | 5.28 (1.54) | 4.54 (1.55) | <0.001 |
| Missing | 12 (4.5%) | 12 (2.0%) | 86 (2.1%) |  |
| **Vaccine factor 4** |  |  |  |  |
| Mean (SD) | 3.69 (1.92) | 4.23 (1.59) | 4.12 (1.73) | <0.001 |
| Missing | 12 (4.5%) | 11 (1.8%) | 60 (1.5%) |  |
| **Vaccine factor 5** |  |  |  |  |
| Mean (SD) | 4.43 (1.77) | 4.64 (1.48) | 5.55 (1.45) | <0.001 |
| Missing | 10 (3.8%) | 11 (1.8%) | 57 (1.4%) |  |
| **Vaccine factor 6** |  |  |  |  |
| Mean (SD) | 5.30 (1.74) | 4.88 (1.44) | 4.84 (1.56) | <0.001 |
| Missing | 15 (5.6%) | 16 (2.6%) | 88 (2.2%) |  |
| **Vaccine factor 7** |  |  |  |  |
| Mean (SD) | 3.92 (1.83) | 4.09 (1.59) | 4.05 (1.74) | 0.275 |
| Missing | 14 (5.3%) | 14 (2.3%) | 69 (1.7%) |  |
| **Vaccine factor 8** |  |  |  |  |
| Mean (SD) | 3.65 (1.99) | 3.75 (1.74) | 3.56 (1.71) | 0.077 |
| Missing | 17 (6.4%) | 15 (2.5%) | 92 (2.3%) |  |
| Note. SD: standard deviation | | | | |

Table 3. Cronbach’s alphas and McDonald’s Omegas for relevant measures, at T1 and T2 respectively (N = 2695)

|  | **T1** | | **T2** | |
| --- | --- | --- | --- | --- |
| **Measure** | **Cronbach’s alpha** | **McDonald’s Omega** | **Cronbach’s alpha** | **McDonald’s Omega** |
| **Psychological Distress** | 0.96 | 0.96 | 0.96 | 0.96 |
| **Perceived social support** | 0.92 | 0.91 | 0.92 | 0.92 |
| **Attitudes towards vaccines** | 0.92 | 0.92 | 0.93 | 0.93 |
